# Puerarin Suppresses Proliferation of Endometriotic Stromal Cells Partly via the MAPK Signaling Pathway Induced by 17ß-estradiol-BSA

**DOI:** 10.1371/journal.pone.0045529

**Published:** 2012-09-19

**Authors:** Wen Cheng, Lizao Chen, Shengsheng Yang, Jie Han, Dongxia Zhai, Jian Ni, Chaoqin Yu, Zailong Cai

**Affiliations:** 1 Department of Traditional Chinese Medicine, Changhai Hospital and Department of Biochemistry and Molecular Biology, Second Military Medical University, Shanghai, China; 2 Institute of Micro/Nano Science and Technology, Shanghai Jiaotong University, Shanghai, China; Universidade Federal do Rio de Janeiro, Brazil

## Abstract

**Background:**

Puerarin is a major isoflavonoid compound extracted from *Radix puerariae*. It has a weak estrogenic action by binding to estrogen receptors (ERs). In our early clinical practice to treat endometriosis, a better therapeutic effect was achieved if the formula of traditional Chinese medicine included *Radix puerariae*. The genomic and non-genomic effects of puerarin were studied in our Lab. This study aims to investigate the ability of puerarin to bind competitively to ERs in human endometriotic stromal cells (ESCs), determine whether and how puerarin may influence phosphorylation of the non-genomic signaling pathway induced by 17ß-estradiol conjugated to BSA (E_2_-BSA).

**Methodology:**

ESCs were successfully established. Binding of puerarin to ERs was assessed by a radioactive competitive binding assay in ESCs. Activation of the signaling pathway was screened by human phospho-kinase array, and was further confirmed by western blot. Cell proliferation was analyzed according to the protocol of CCK-8. The mRNA and protein levels of cyclin D1, Cox-2 and Cyp19 were determined by real-time PCR and western blotting. Inhibitor of MEK1/2 or ER antagonist was used to confirm the involved signal pathway.

**Principal Findings:**

Our data demonstrated that the total binding ability of puerarin to ERs on viable cells is around 1/3 that of 17ß-estradiol (E_2_). E_2_-BSA was able to trigger a rapid, non-genomic, membrane-mediated activation of ERK1/2 in ESCs and this phenomenon was associated with an increased proliferation of ESCs. Treating ESCs with puerarin abrogated the phosphorylation of ERK and significantly decreased cell proliferation, as well as related gene expression levels enhanced by E_2_-BSA.

**Conclusions/Significance:**

Puerarin suppresses proliferation of ESCs induced by E_2_-BSA partly via impeding a rapid, non-genomic, membrane-initiated ERK pathway, and down-regulation of Cyclin D1, Cox-2 and Cyp19 are involved in the process. Our data further show that puerarin may be a new candidate to treat endometriosis.

## Introduction

Endometriosis (EM) is an estrogen-dependent benign disease characterized by extra-uterine implantation and ectopic growth of endometrium. It affects 10–20% of women in their reproductive years, causing chronic pelvic pain and infertility [1]. Current treatments, based on hormonal therapy or surgery, are often insufficient [2]–[4]. Therefore, exploring novel therapeutic strategies is necessary for improving the clinical management of patients with endometriosis.

17ß-estradiol (E_2_), which can be locally and distantly secreted by endometriotic cells and ovary, plays a critical role in the development and progression of endometriosis, in which it acts as an important regulator of cell proliferation, survival and differentiation. Clinical and experimental data have clearly established that E_2_ is the key factor leading to adhesion, aggression and angiogenesis in the pathological process of endometriosis [5]. The predominant physiological effects of E_2_ traditionally have been considered to be based on interaction of E_2_ with estrogen receptors (ERα and ERß). The effects are brought about via the regulation of transcriptional processes, involving nuclear translocation, binding to specific estrogen response elements (EREs) and ultimately regulation of gene expression [6]. However, a number of studies have shown that steroid hormones, particularly E_2_, are able to elicit cellular responses that are too rapid to be mediated by the activation of transcription and transduction mechanisms. Many rapid effects of estrogen, with an onset in seconds, have been documented in numerous tissues, and the time courses of these events, which are similar to those elicited by growth factors and peptide hormones, suggest that they do not require precedent gene transcription or protein synthesis [7]. Rather, these rapid non-genomic effects of estrogen may be due to activation of membrane-associated signaling pathways, including protein kinase A, phosphotidylinositol-3 kinase and mitogen-activated protein kinase (MAPK) signaling pathways [8]–[10].

Puerarin is a major isoflavonoid compound extracted from the Chinese medicinal herb, *Radix puerariae*. This compound has been suggested to be useful in the management of various disorders, such as coronary artery disease, liver fibrosis, neurotoxicity and bone injury [11]–[14].

Puerarin, a phytoestrogen with a weak estrogenic action, binds to estrogen receptors, thereby competing with E_2_ and producing an anti-estrogenic effect. In our early clinical practice to treat endometriosis, a better therapeutic effect was achieved if the formula of traditional Chinese medicine included *Radix puerariae*. Subsequently, the effects of puerarin to treat rat models of endometriosis, and to suppress invasion and vascularization of endometriotic tissue stimulated by E_2_ were elucidated in our laboratory [5], [15]. However, the underlying molecular mechanisms mediating such actions of puerarin are still to be further characterized, including the ability of puerarin to bind to ERs and non-genomic effects of puerarin. Therefore, the main aims of the present study were to determine the ability of puerarin to bind to ERs, and to investigate the non-genomic effects of puerarin and its downstream signaling pathway.

## Results

### Estrogen Receptor Competitive Binding Assay

**Figure 1:**
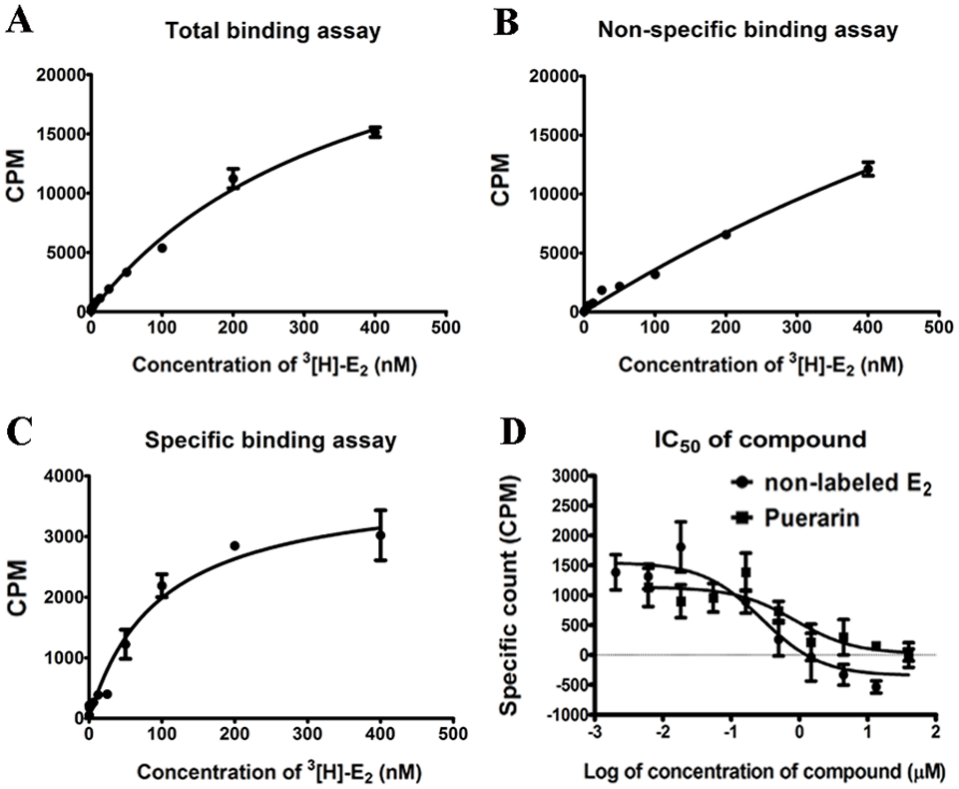
Estrogen receptor competitive binding assay. ESCs were incubated in the presence of E_2_ or puerarin solutions for 1 h. After removal of the medium, [^3^H]-E_2_ binding capacity was measured. (A) Total binding of [^3^H]-E_2_ to ERs in ESCs; the concentrations of [^3^H]-E_2_ used were 0, 0.1950, 0.3906, 0.7813, 1.5625, 3.125, 6.25, 12.5, 25, 50, 100, 200 and 400 nmol/L. (B) Non-specific binding of [^3^H]-E_2_. (C) Specific binding of [^3^H]-E_2_, calculated by subtracting the non-specific binding values from the total binding values. (D) Measurement of [^3^H]-E_2_ binding to ERs in the presence of varying concentrations of E_2_ (0, 0.0061, 0.0183, 0.0549, 0.1646, 0.4938, 1.4815, 4.4444, 13.3333 and 40 µmol/L) or puerarin (0, 0.0061, 0.0183, 0.0549, 0.1646, 0.4938, 1.4815, 4.4444, 13.3333 and 40 µmol/L). The figures represent data from three experiments; each experiment was performed in triplicate, and data are represented as the mean ± SD.

To test the ability of puerarin and E_2_ to bind competitively to ERs, ESCs were isolated and cultured, so that the purity of ESCs amongst the isolated cells was around 95% [5]. The radioactivity count was measured in the absence (for the total binding assay) (Fig. 1A) or in the presence (for the non-specific binding assay) of a 100-fold molar excess of unlabeled E_2_ (Fig. 1B). The Kd value determined from the above two assays was 96 nmol/L (Fig. 1C). In the subsequent experiments, specific binding of [^3^H]-E_2_ to ERs was competed by increasing concentrations of unlabeled E_2_, and the radioactivity value decreased in a concentration-dependent manner. The IC_50_ value for E_2_ was found to be 0.28 µmol/L, which was calculated by using Graphpad Prism 5.0 software. Over the same range of concentrations as unlabeled E_2_, puerarin also competed against the specific binding of [^3^H]-E_2_ to ERs, and the IC_50_ of puerarin was determined to be 0.87 µmol/L (Fig. 1D). The RBA calculated from the assays was 32.2%, suggesting that a direct interaction exists between ERs and puerarin, and that the binding ability of puerarin is one third that of E_2_.

### ERK1/2 was Highly Activated by E_2_-BSA, which was Reversed by Puerarin

**Figure 2:**
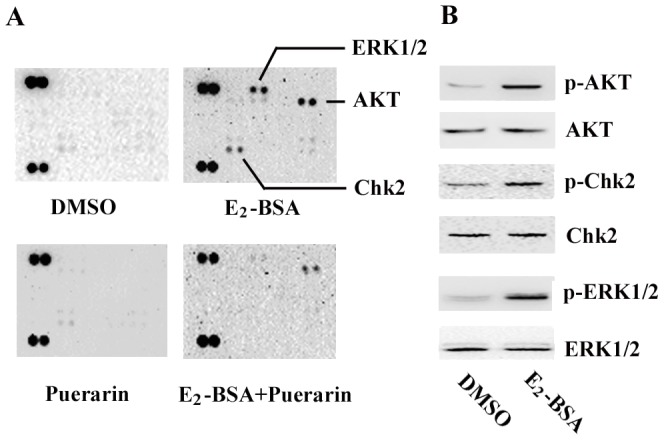
Phospho-proteomic profiling of E_2_-BSA and puerarin effects on ERK1/2 phosphorylation in ESCs and confirmed by western blot analysis. Cells were treated as indicated. A. AKT, Chk2 and ERK1/2 were activated by E_2_-BSA and suppressed by puerarin. B. Western blot analysis of E_2_-BSA on phosphorylation in ESCs. Cells were treated with vehicle (DMSO) or E_2_-BSA (10^−8^ mol/L) for 15 min. AKT, Chk2 and ERK1/2 were activated by E_2_-BSA. Expression was normalized to total ERK1/2.

To discover whether a non-genomic pathway could be activated by E_2_-BSA and whether it could be reversed by puerarin, ESCs were treated with DMSO vehicle or E_2_-BSA or E_2_-BSA plus puerarin or puerarin alone, and human phospho-kinase array assays were performed. As shown in Fig. 2A, ERK1/2, AKT and Chk2 were activated in the E_2_-BSA treatment group, as compared with the DMSO treatment group, and high levels of phosphorylation of all three were further confirmed by western blot (Fig. 2B). Interestingly, the phosphorylations were sharply reduced by treatment with E_2_-BSA combining puerarin (Fig. 2A), as compared with the E_2_-BSA treatment group. Since the activation of ERK1/2 was a novel finding and had a significant change in this setting, we focused on ERK1/2 in the following experiments.

**Figure 3:**
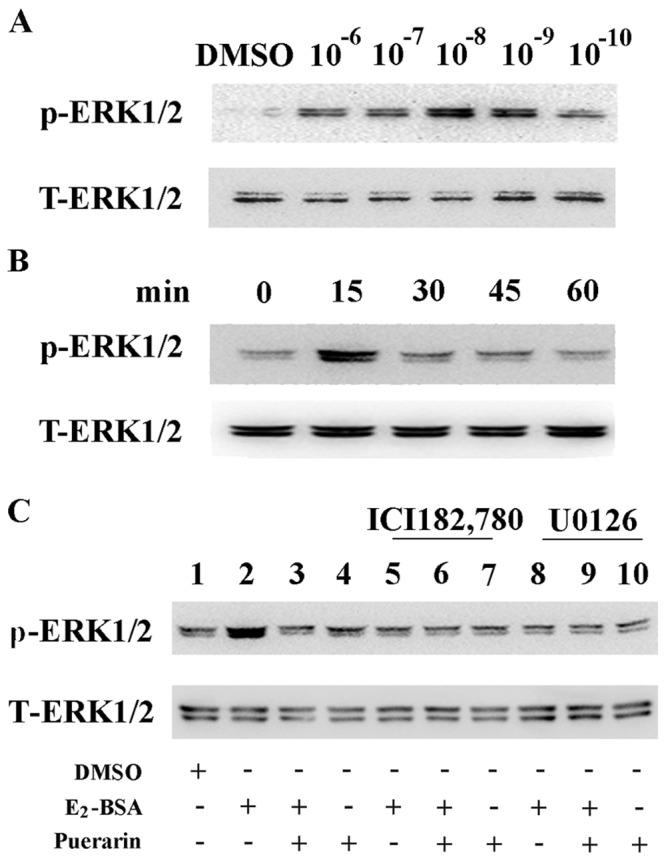
Western blot analysis of E_2_-BSA and puerarin effects on ERK1/2 phosphorylation in ESCs. Cells were treated as indicated. A. Concentration-dependent effects of E_2_-BSA on ERK1/2 phosphorylation in ESCs. Cells were treated with vehicle (DMSO) or E_2_-BSA (10^−10^ to 10^−6^ mol/L) for 15 min. B. Time-dependent effects of E_2_-BSA on ERK1/2 phosphorylation in ESCs. Cells were treated with 10^−8^ mol/L of E_2_-BSA for 0, 15, 30, 45 or 60 min. C. ESCs were treated with 10^−8^ mol/L of E_2_-BSA, 10^−9^ mol/L puerarin or both, as indicated; in some groups, cells were pretreated with 20 µmol/L of U0126 or 1 µmol/L of ICI182,780 for 30 min prior to the stimulation. Expression was normalized to total ERK1/2.

The ESCs were treated with either vehicle or a range of concentrations of E_2_-BSA (10^−10^ to 10^−6^ mol/L) for 15 min to determine the most effective concentration to cause ERK phosphorylation. Western blotting showed that E_2_-BSA increased the levels of p-ERK1/2 at all concentrations tested. However, the most effective concentration of E_2_-BSA was 10^−8^ mol/L, which induced the highest increase in p-ERK1/2 levels, to a level that was about fivefold greater than that seen in control cells (Fig. 3A).

To determine the time course of this E_2_-BSA action, ESCs were treated with E_2_-BSA (10^−8^ mol/L) for 0, 15, 30, 45 and 60 min. E_2_-BSA was able to increase ERK1/2 phosphorylation, reaching a peak at 15 min before declining sharply to baseline levels within 1 h (Fig. 3B).

To assess the effect of puerarin on the induction of ERK1/2 phosphorylation by E_2_-BSA, ESCs were treated with puerarin (10^−9^ mol/L), with or without E_2_-BSA, for 15 min. Fig. 3C shows that puerarin decreased the activating effect of E_2_-BSA on ERK1/2 phosphorylation.

To further identify any potential non-classical ER involvement in the induction of ERK1/2 phosphorylation by E_2_-BSA, the above experiments were repeated in ESCs that had been pretreated with the ER antagonist, ICI182,780. The results showed that pretreatment with ICI182,780 inhibited the effect of E_2_-BSA on ERK1/2 phosphorylation (Fig. 3C), suggesting that this phosphorylation effect is mediated by the plasma membrane ERs (mER).

To investigate the effect of a MAPK/ERK kinases (MEK) inhibitor on the induction of ERK1/2 phosphorylation by E_2_-BSA, cells were pretreated with U0126 (20 µmol/L) for 30 min, and then E_2_-BSA alone, E_2_-BSA plus puerarin, or puerarin alone, were added to the cultures for 15 min. Pretreatment with U0126 abolished ERK phosphorylation elicited by E_2_-BSA (Fig. 3C), suggesting that ERK phosphorylation in the presence of E_2_-BSA is MEK-dependent.

### E_2_-BSA Induces the Proliferation of ESCs, which was Reversed by Puerarin

**Figure 4:**
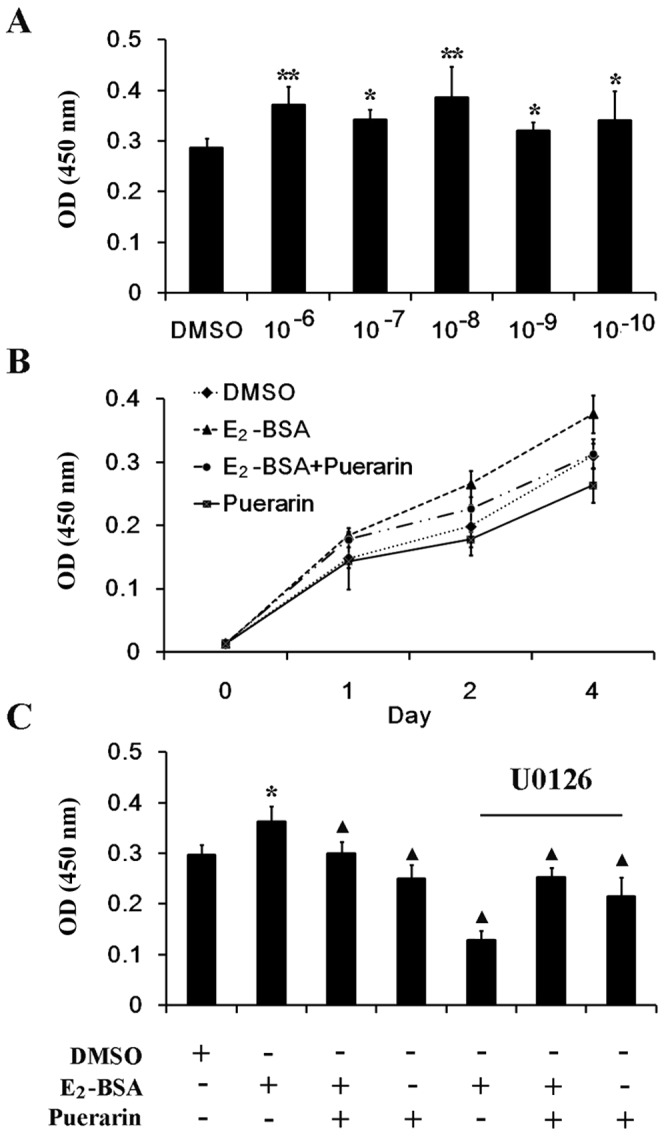
Effect of E_2_-BSA and puerarin on cell proliferation in ESCs. (A) ESCs were treated with various concentrations (10^−10^ to 10^−6^ mol/L) of E_2_-BSA for 4 d. (B) Growth curves of ESCs incubated with E_2_-BSA (10^−8^ mol/L) and/or puerarin (10^−9^ mol/L) for 0, 1, 2, 4 d. (C) Cells were treated for 4 d with E_2_-BSA (10^−8^ mol/L) in the absence or presence of puerarin (10^−9^ mol/L) and/or pretreated with U0126 (20 µmol/L) for 60 min. The figures represent data obtained from three experiments; each experiment was performed in triplicate, and data are represented as the mean ± SD. DMSO treatment was used as the vehicle control. *Vs. control*,**, *P*<*0.01,* *, *P*<*0.05; vs. E_2_-BSA, ▴, P*<*0.05.*

To find out whether E_2_-BSA could induce proliferation of ESCs, and whether this induction could be reversed by puerarin, we carried out cell proliferation experiments. As shown in Fig. 4A, when compared with vehicle control, ESCs treated with the indicated concentrations of E_2_-BSA for 4 d demonstrated a significant increase in cell proliferation, with the maximal effect seen at a concentration of 10^−8 ^mol/L E_2_-BSA (*P*<0.01).

We then investigated the potential effect of puerarin on cell proliferation promoted by 10^−8 ^mol/L E_2_-BSA. Compared with the E_2_-BSA group, cell proliferation decreased when E_2_-BSA treatment was combined with puerarin. In addition, puerarin alone was found to suppress cell growth slightly but not statistically significant (*P*>0.05), as compared with the DMSO group (Fig. 4B).

This effect of E_2_-BSA to stimulate cell proliferation was significantly reduced by pretreatment with U0126 for 60 min, suggesting that the ERK pathway is involved in this process (Fig. 4C).

### Puerarin Suppresses Related Gene Expression Induced by E_2_-BSA in ESCs

**Figure 5:**
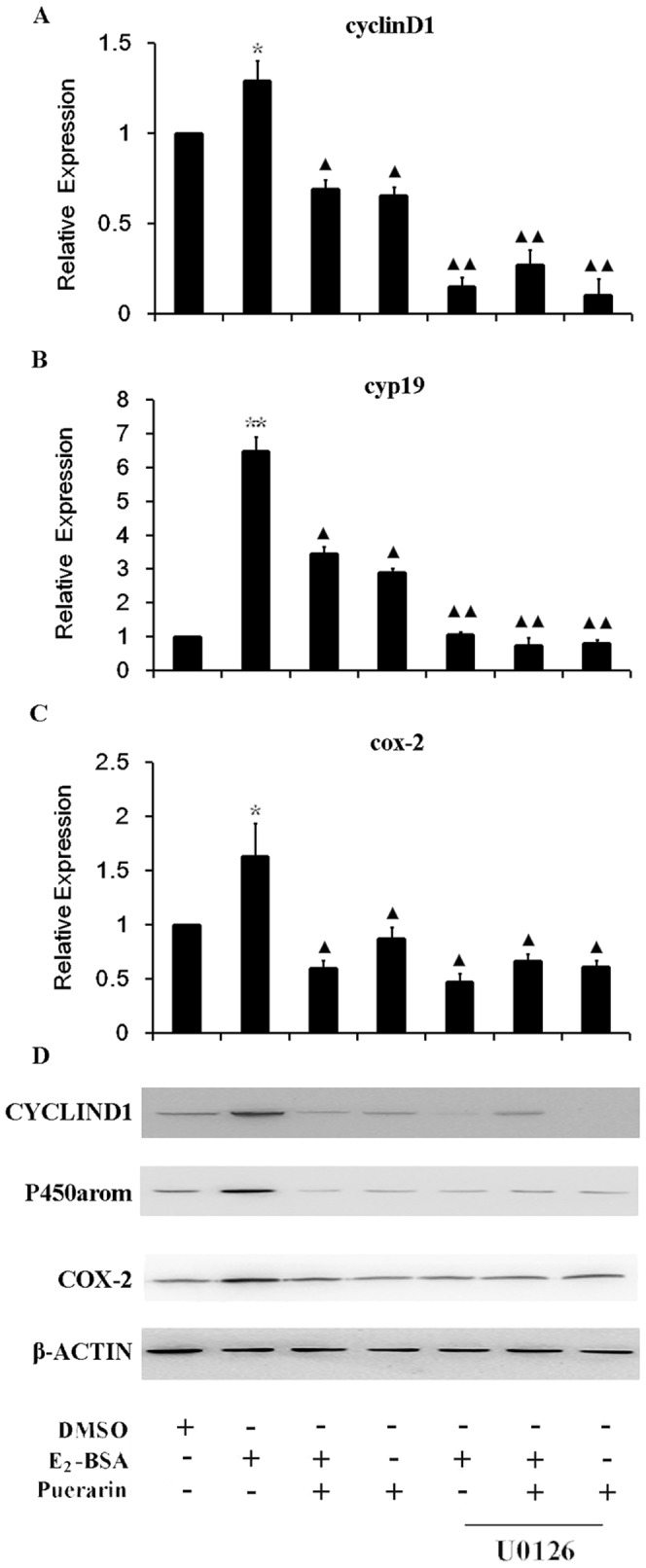
Analysis of expression of cyclin D1, cyp19 and cox-2 genes in ESCs subjected to various treatments. ESCs were treated with E_2_-BSA, puerarin or both for 24 h, in the absence and presence of pretreatment with U0126 for 60 min. Total RNA was isolated, RT quantitative real-time PCR was performed and relative expression of cyclin D1 (A), cyp19 (B) and cox-2 (C) were obtained. GAPDH served as an internal control. Individual bars show relative mRNA levels expressed as the mean ± S.E.M., determined from three separate assays. Protein levels were detected by western blotting (D). Group *vs.* control, **, *P*<*0.01 and* *, *P*<*0.05;* Group *vs.* E_2_-BSA, *▴▴, P*<*0.01* and *▴, P*<*0.05*.

Cyclin D1 serves as a key sensor and integrator of extracellular signals in cells in the early-to-mid G1 phase of cell proliferation and differentiation [16]. Cyclooxygenase-2 (COX-2) is a key enzyme in the biosynthesis of prostaglandin E2 (PGE2), which is believed to be implicated in the development of endometriosis and its symptoms [17]. The aromatase P450 (P450arom), which is encoded by the cyp19 gene, is a key enzyme for biosynthesis of estrogen, and is stimulated by PGE2. This results in local production of estrogen, which induces PGE2 formation and establishes a positive feedback cycle, becoming one of the important factors in the pathogenesis of endometriosis [18]. To assess the effects of E_2_-BSA on expression of these genes, the levels of the mRNA and protein products of the above genes were detected in ESCs subjected to the treatments indicated in Fig. 5.

Our results showed that E_2_-BSA could induce cyclin D1 mRNA expression, and that this effect was suppressed by puerarin. Furthermore, when ESCs were pretreated with U0126, the effect of E_2_-BSA on cyclin D1 mRNA levels was considerably reduced (Fig. 5A) and puerarin couldn’t suppress the mRNA level any more. In addition, cyclin D1 protein levels, detected by western blotting, were found to correlate with mRNA levels (Fig. 5D). Similar results were obtained with cyp19 (Fig. 5B) and cox-2 (Fig. 5C), showing that the effects of E_2_-BSA and puerarin on cyclin D1, cyp19 and cox-2 mRNA expression and protein levels are ERK dependent.

## Discussion

The current standard medical treatments for endometriosis include GnRH agonists, contraceptive steroids, progestogens, and androgens [4], [19]–[20], all of which aim to lower circulating E_2_ concentrations. Of these agents, GnRH agonists appear to be the most effective, but they are expensive, and long-term treatment is not possible because of the loss of bone mineral density. However, puerarin is cheap, and it can be used for long periods without severe side effects; therefore, medical treatment with this agent is a good option for avoiding disease relapse after the initial surgical and/or medical therapy.

To further elucidate the general mechanism of puerarin action against estrogen, the experiments were carried out on ESCs which were from kinds of patients with no specificity. First, we focused on the ability of puerarin to bind to ERs on viable cells, so the whole cell ER competitive binding assay was performed. This assay has the advantage of providing the means to rapidly and reproducibly quantitate puerarin and E_2_ specific binding in small samples of whole cells in culture [21] and it also imitates the local circumstance in vivo. The results showed that puerarin has the ability to compete with estrogen at the ERs on whole ESCs.

In estrogen-dependent disease, the classical pathway by which estrogen induces cell proliferation is through binding to a ligand-dependent ER, thus inducing its conformational change and subsequently interacting with EREs, together with multiprotein complexes of transcription factors and co-activators, to activate transcription [22].

In addition to this classic genomic effect of estrogen, several non-genomic actions have also been reported that initiate at the cell surface through binding of estrogen to membrane receptors [23], including calcium mobilization [24]–[25], cAMP stimulation [26], phospholipase C activation and inositol phosphate generation [27], and MAPK activation [28]. Since puerarin has the ability to compete with estrogen for the ERs, it was of interest to us to ascertain whether puerarin could antagonize the effect of estrogen to cause proliferation through rapid non-genomic pathways.

To distinguish non-transcriptional signaling pathways, E_2_-BSA, a macromolecular complex considered to be membrane-impermeable, was used, since this is prevented from reaching the classical ERs in the nucleus [29]–[31]. Human phospho-kinase array experiments showed that E_2_-BSA induced significant phosphorylation of several kinases, including AKT, Chk2 and ERK1/2, whereas puerarin weakened its effect. By western blotting, we confirmed that E_2_-BSA-induced activation of ERK1/2 in ESCs was transient, reaching a peak at 15 min before returning to basal levels within an hour. This observation allows us to propose that this stimulating phenomenon initiates at the plasma membrane, and does not involve ER-mediated gene transcription.

ERK, one of three well-characterized subfamilies of MAPK, phosphorylates specific tyrosine and threonine residues of target protein substrates, and regulates cellular activities that include gene expression, mitosis, movement, metabolism, survival, and programmed cell death [32]–[33]. Our data that puerarin reduced ERK1/2 phosphorylation in ESCs treated with E_2_-BSA support our proposition that puerarin could exhibit anti-estrogenic activity partly through inhibiting of the ERKs signaling pathway. Further evidence that membrane receptors are involved in the non-genomic effects of E_2_-BSA is provided by the observation that when ESCs were treated with the ER antagonist, ICI182,780, phosphorylation of ERK1/2 was inhibited. Meantime, puerarin can’t reverse the phosphorylation of ERK1/2 on this occasion. These phenomenons indicated that puerarin inhibited activation of ERK through competing with estrogen to bind to membrane receptors initiated MAPK signaling (Fig. 3C).

We found that all the doses of E_2_-BSA we tested could promote proliferation of ESCs as shown in figure 4A and various concentraions (from 10^−10^ mol/L to 10^−4^ mol/L) of puerarin could reverse this effect by CCK-8 assay and that 10^−9^ mol/L achieved the most useful effect (data not shown). When U0126, a “small molecule” inhibitor of MEK1/2, the upstream activating kinases of ERK1/2, was added, ESCs proliferation couldn’t be promoted by E_2_-BSA anymore, and even the proliferation was much slower than the DMSO treatment group (Fig. 4C). These results demonstrated that E_2_-BSA promoted ESCs proliferation partly through activating ERK1/2 signaling pathway. Unexpectedly, puerarin was shown to be able to partially rescue inhibiting effects of the kinase inhibitor. This result suggested that when ESCs was pretreated with U0126, non-genomic ERK pathway of E_2_-BSA and puerarin was blocked, however, genomic effects of puerarin were still remained and played a weak estrogenic role (Fig. 3C).

Some literatures documented that expression of endometriosis-related genes; cyclin D1, cyp19 and cox-2 depend on non-genomic ERK pathway [16], [34]–[35]. In addition, we found that expression of these genes, as well as phosphorylation of ERK1/2 was completely inhibited by U0126 in ESCs. This supports the conclusion that the ERK1/2 signaling pathway plays an important role in the expression of these genes which are related to cell proliferation and puerarin could suppress these key genes expression by inhibiting ERK1/2 activation.

In conclusion, the total binding ability of puerarin to ERs on viable ESCs is around 1/3 that of E_2_, puerarin suppresses proliferation of ESCs induced by E_2_-BSA, partly via impeding a rapid, non-genomic, membrane-initiated ERK pathway, and down-regulation of cyclin D1, cox-2 and cyp19 are involved in this process. Puerarin may represent a potentially new approach in the medical treatment of endometriosis.

## Materials and Methods

### Sample Collection and Cell Isolation

Tissues were obtained from patients, with ovarian endometriotic cysts, undergoing laparoscopy. For all patients included in this study, pathology analysis was carried out on one part of the biopsy to confirm the presence of endometriotic tissue. The mean age of the subjects was 37.6±5.8 years (range 27–49); all subjects had regular menstrual cycles and had been independent of hormonal treatment for at least 3 months before surgery. The study was approved by the Committee on Human Research of Changhai Hospital, and written informed consent was obtained from all subjects.

Specimens were collected, and ESCs were purified, cultured and identified as previously described [36].

### Competitive Estrogen Receptor Whole ESCs Binding Assay

ESCs were seeded at a density of 2×10^4^ cells/well in 96-well plates with phenol red-free DMEM (Dulbecco’s modified Eagle’s medium; PAA, Linz, Austria) supplemented with 10% charcoal-stripped fetal bovine serum (CSFBS) (Biological Industries, Israel). After cultivation at 37°C in a 5% CO_2_ atmosphere for 4 d, competitive binding assays were performed on whole cells using a previously described method [37]. The dissociation constant (K_d_) of the ER and [2,4,6,7^−3^H]-Estradiol ([^3^H]-E_2_; PerkinElmer, Massachusetts, USA) complex was measured with nonlinear regression techniques. We then used the K_d_ value of labeled E_2_ to determine the IC_50_ values for E_2_ (Sigma Chemical Co., St Louis, Mo, USA) and puerarin (Sigma Chemical Co., St Louis, Mo, USA), i.e. the concentrations of E_2_ and puerarin at which half of the specific binding of [^3^H]-E_2_ had been inhibited. The relative binding affinity (RBA) was calculated as: ((IC_50_) E_2_/(IC_50_) puerarin) ×100%. RBA data was determined from the mean of three independent experiments, and each was performed in triplicate.

### Phospho-kinase Proteome Profiling

ESCs were plated in 100 mm tissue culture dishes, and cultured in DMEM supplemented with 10% FBS until they reached 70% confluence. After serum starvation for 24 h, cells were then cultured in phenol red-free DMEM supplemented with 10% CSFBS with or without 10^−8^ mol/L of E_2_-BSA and/or puerarin (10^−9^ mol/L) (Sigma Chemical Co., St Louis, Mo, USA) for 20 min. Cells were then processed, following the human phospho-kinase array kit (Proteome Profiler™; R&D Systems, Minneapolis, USA) instructions. Phospho-kinase array data were developed on X-ray films following exposure to chemiluminescent reagents.

### Western Blot Analysis

ESCs were seeded in 40 mm dishes at a density of 3×10^6^ cells per dish. After serum starvation for 24 h, cells were treated with various doses of E_2_-BSA (Sigma Chemical Co., St Louis, Mo, USA ) (10^−10^ to 10^−6^ mol/L) or dimethyl sulfoxide (DMSO) for 15 min, or by E_2_-BSA (10^−8^ mol/L) for various times as indicated. In addition, cells were exposed to 10^−9^ mol/L of puerarin with or without E_2_-BSA (10^−8^ mol/L). When inhibitors were applied, cells were pre-incubated with the ER antagonist, ICI182,780 (Sigma Chemical Co., St Louis, Mo, USA) (1 µmol/L) for 30 min, or with U0126 (Cell Signaling Technology, Beverly, Massachusetts, USA) (20 µmol/L) for 1 h. Cells were lysed, and western blotted as previously described [38]. Data S1 lists the antibodies used in this research.

### Cell Proliferation

For cell proliferation studies, ESCs were seeded in 96-well plates (1×10^4^ cells/well) and maintained in conditions as described above. After serum starvation, the cells were further treated with the indicated compounds. The proliferative potential of cells was analyzed according to the protocol of CCK-8 (Dojindo, Japan).

### Reverse Transcription Reaction and Quantitative Real-time RT-PCR

The ESCs (1×10^6^ cells per well) were cultured in 40 mm dishes and treated as described previously. After 24 h culture with 10^−9^ mol/L of puerarin with or without E_2_-BSA (10^−8^ mol/L), and in the presence or absence of U0126 (20 µmol/L) for 1 h, the total cell RNA in each culture was extracted and quantitative real-time RT-PCR was performed as described previously [38]. Primer sequences are shown in data S1. All samples were examined in triplicate and included no-template controls.

### Statistical Analysis

Data were first assessed by tests of normality and homogeneity of variances, and then were represented as means ± SD, calculated from three independent experiments. Statistical comparisons between groups were performed using one-way ANOVA followed by post-hoc multiple comparisons where appropriate. A *P* value <0.05 was considered to indicate statistical significance.

## Supporting Information

Data S1 **Antibodies and Primers used in this research.**
(DOCX)
