# Supplementary material for: Puerarin Suppresses Proliferation of Endometriotic Stromal Cells Partly via the MAPK Signaling Pathway Induced by 17ß-estradiol-BSA
Source: PLoS One. 2012 Sep 19;7(9):e45529. doi: 10.1371/journal.pone.0045529 (PMC3446905; doi:10.1371/journal.pone.0045529)
Supplement: Data S1 — Antibodies and Primers used in this research. (DOCX) [file pone.0045529.s001.docx]

**Data S1: Antibodies and Primers used in this research:**

| **Antibody** | | **Host species** | **Molecular weight (KD)** | | **Source** |
| --- | --- | --- | --- | --- | --- |
| phospho-ERK1/ERK2 (Thr202/Tyr204) | | Rabbit | 42,44 | | Cell Signaling Technology |
| ERK1/ERK2 (Thr202/Tyr204) | | Rabbit | 42,44 | | Cell Signaling Technology |
| phospho-AKT  (Ser473) | | Rabbit | 60 | | Cell Signaling Technology |
| AKT | | Rabbit | 60 | | Cell Signaling Technology |
| phospho-Chk2  (Thr68) | | Rabbit | 62 | | Cell Signaling Technology |
| Chk2 | | Rabbit | 62 | | Cell Signaling Technology |
| COX-2 | | Rabbit | 74 | | Cell Signaling Technology |
| Aromatase P450 | | Rabbit | 53 | | Abcam |
| Cyclin D1 | | Rabbit | 33 | | Abcam |
| ß-actin | | Mouse | 45 | | Sigma |
| Goat anti-rabbit IgG | | | | | Cell Signaling Technology |
| Goat anti-mouse IgG | | | | | Abcam |
| **Primers** | **Forward primer sequence (5'-3')** | | | **Reverse primer sequence (5'-3')** | |
| cyp19 | AGTGCATCGGTATGCATGAG | | | AGAAGGGTCAACACGTCCAC | |
| cox-2 | ACGGTTTGCTGTGGGGCAGG | | | CCCGCAGCCAGATTGTGGCA | |
| cyclin D1 | GCCCGAGGAGCTGCTGCAAAT | | | TGCCACCATGGAGGGCGGAT | |
| GAPDH | CGGAGTCAACGGATTTGGTCGTATTGG | | | GCTCCTGGAAGATGGTGATGGGATTTCC | |
